# Xanthatin induces apoptosis through ROS-mediated c-FLIP inhibition in human retinoblastoma cells

**DOI:** 10.3389/fmed.2025.1554934

**Published:** 2025-04-16

**Authors:** Xue Gao, Yixiao Li, Haoran Xu, Shouxiang Ni, Hong Pan, Chunli Ma, Xiaofei Zhao, Han Zhang

**Affiliations:** ^1^Department of Ophthalmology, The Second Hospital of Shandong University, Jinan, China; ^2^Department of Ophthalmology, Shandong Provincial Hospital, Cheeloo College of Medicine, Shandong University, Jinan, China; ^3^Department of Ophthalmology, Shandong Provincial Hospital Affiliated to Shandong First Medical University, Jinan, China; ^4^Shandong First Medical University & Shandong Academy of Medical Sciences, Jinan, China

**Keywords:** retinoblastoma, xanthatin, apoptosis, reactive oxygen species, CASP8 and FADD-like apoptosis regulating protein

## Abstract

Retinoblastoma is widely considered the most frequent primary intraocular malignancy during childhood. Xanthatin has been reported to selectively inhibit the proliferation of RB cells, but the underlying mechanism remains uncertain. In this study, human RB cells were treated with different doses of xanthatin, and then cell survival, cell apoptosis, and protein expression were assessed using CCK8 assays, flow cytometry, and western blotting to investigate the possible mechanism of xanthatin in RB cells. A human RB xenograft model was established to demonstrate the effect of xanthatin *in vivo*. Our study shows that xanthatin inhibited cell survival and induced apoptosis in human RB cells. Moreover, xanthatin induced the downregulation of CASP8 and FADD-like apoptosis regulating protein (c-FLIP) and increased the cleavage of caspase-8, caspase-9, caspase-3, and PARP. c-FLIP overexpression impaired xanthatin-induced apoptosis. Furthermore, NAC, which can reduce xanthatin-triggered Reactive oxygen species (ROS), alleviated xanthin-induced apoptosis and c-FLIP downregulation. *In vivo*, analysis confirmed that xanthatin was an efficacious drug against xenograft tumors. Xanthatin induced apoptosis of the human RB cells both *in vivo* and *in vitro* through ROS-mediated c-FLIP inhibition. Our research provides important mechanistic insight into potential cancer treatments with ROS/c-FLIP axis in xanthatin-induced apoptosis and makes them candidates for developing new directed therapies.

## 1 Introduction

Retinoblastoma (RB), a primary pediatric intraocular cancer, is the third most prevalent childhood tumor and affects the health of children worldwide (1). The clinical management of RB is still challenging. Current treatments for RB include enucleation, radiation therapy, and systemic chemotherapy (2). In developing countries, owing to medical, economic, and social constraints, many children are diagnosed in the middle or late stages, leading to extremely high rates of blindness and disability (3).

*Xanthium strumarium* L. (Asteraceae) is a well-known traditional herbal medicine in China and Vietnam (4, 5). The fruits and roots of this plant are utilized as an anti-inflammatory herb to treat ailments such as nasal sinusitis and arthritis (6), and the whole plant is used to treat bacterial infections, diabetes, skin itchiness, and inflammatory diseases (7). Recently, the antitumor activity of this plant has been confirmed in studies on various tumors, such as non-small cell lung cancer (8), hepatocellular carcinoma (9), glioma (10), and breast cancer (11). Notably, xanthatin has been shown to selectively inhibit the proliferation of retinoblastoma cells by inducing cell cycle arrest and promoting apoptosis. However, the underlying mechanism and the actual effect *in vivo* require further research (12).

Apoptosis has been recognized as a type of programmed cell death that plays an important role in the genesis of numerous physiological and pathological diseases (13). Cell survival and death are intricately governed by apoptosis which is vital in facilitating embryonic development and maintaining immunological functioning tissue homeostasis (14). Deregulation of this pathway (excessive or recessive) is central to various diseases, such as neurodegenerative diseases, ischaemic damage, autoimmune disorders, infectious diseases, and many types of cancer (15, 16). Caspase-8 is an initiator caspase that plays a crucial role in the extrinsic apoptotic pathway (also known as the death receptor pathway). When death receptors on the cell surface (such as Fas/CD95 or TRAIL receptors) are activated by their ligands, a death-inducing signaling complex (DISC) is formed. Caspase-8 is recruited to the DISC and becomes activated. Activated caspase-8 can then activate downstream executioner caspases, such as caspase-3, which cleave various intracellular substrates, ultimately leading to apoptosis.

Cellular FADD-like IL-1β-converting enzyme (FLICE)-inhibitory protein (c-FLIP) is a key antiapoptotic regulator and resistance factor that inhibits cell death mediated by the death receptors Fas-L, DR4, DR5, and TNF-α (17). c-FLIP upregulation has been observed in various kinds of tumors, and c-FLIP downregulation is related to apoptosis caused by various cytokines and chemotherapeutic drugs (17). Therefore, c-FLIP is an important target for cancer therapy (18). It shares structural similarity with caspase-8 and can compete with caspase-8 for binding to the DISC, thereby inhibiting caspase-8 activation and blocking apoptosis. c-FLIP competes with FADD (Fas-associated death domain protein) for binding, preventing the full activation of caspase-8 within the DISC.

In this scenario, even if caspase-8 expression increases, its ability to effectively activate caspase-3 is hampered by the blocking action of c-FLIP, leading to a decrease in caspase-3 activity and even protein expression. Cancer cells exploit this mechanism to evade the induction of the extrinsic apoptotic pathway, promoting tumor growth and survival. However, whether c-FLIP plays a role in xanthatin-induced apoptosis in retinoblastoma cells and the related mechanism have not been studied.

Reactive oxygen species (ROS), a subproduct of oxidative energy metabolism, are vital in regulating cell signaling, metabolism, and epigenetics. ROS homeostasis is essential for the steady-state functions of cells (19). Under normal conditions, small amounts of ROS are produced and maintained under a dynamic balance governed by antioxidant effectors (20). A slight shift of this balance towards oxidative stress triggers several signaling pathways that cause DNA damage and affect cell functions, such as proliferation, differentiation, migration, and disease initiation and progression (21). An increase in ROS and the downregulation of c-FLIP often co-occur in the apoptosis of many cancer cells (22). However, the effect of ROS on xanthatin-induced apoptosis in retinoblastoma cells and the specific mechanism require further research.

In this study, we investigated the possible mechanism of xanthatin in retinoblastoma and found that xanthatin induced the apoptosis of retinoblastoma cells by elevating ROS and subsequently downregulating the expression of c-FLIP. Our research provides important mechanistic insight into potential cancer treatments with xanthatin and enhances the understanding of human retinoblastoma.

## 2 Materials and methods

### 2.1 Regents

Xanthatin was purchased from ShiZhou Biotechnology, Inc. (Nanjing China SZ2341018). Xanthatin was dissolved with dimethyl sulfoxide (DMSO) to 100 mM and stored at −80°C. NAC was purchased from Beyotime (Shanghai, China, S0077). Antibodies against caspase-8 (Cat No. 9746), caspase-9 (Cat No. 9502), BiP (Cat No. 3183), poly (ADPribose) polymerase (PARP; Cat No. 9542), eIF2α (Cat No. 9722S), IRE1α (Cat No. 3294), and ATF-4 (D4B8) (Cat No. 11815S) were purchased from Cell Signaling Technology (Danvers, MA, United States). An antibody against caspase-3 (Cat No. NB100-56708) was purchased from Imgenex (Novus Biologicals, LLC, Littleton, CO, United States). Antibody against CHOP (Cat No. sc-7351) was purchased from Santa Cruz Biotechnology, Inc. (Dallas, TX, United States). A c-FLIP antibody was purchased from Enzo (ALX-804-961).

### 2.2 Cell lines and cell culture

The Y79 retinoblastoma cell line was obtained from ATCC, and the WERI-RB-1 cell line was obtained from Procell Biotechnology Co., Ltd. (Wuhan, China). The cells were cultured in RPMI-1640 medium (Gibco, United States) supplemented with 10% fetal bovine serum (Gibco, United States), and the cells were incubated at 37°C with 5% CO_2_ and saturated humidity.

### 2.3 CCK8 assay

To determine cell viability, the cells were seeded in 96-well plates at a density of 6.0 × 10^3^ cells per well and were then treated with different concentrations of xanthatin on the second day. The cells were cultured with chemotherapeutics for 24, 36, or 48 h and then subjected to the CCK8 assay. Samples were incubated with 10 μL of CCK8 reagent [Med Chem Express (Monmouth Junction, NJ, United States), HY-k0301] per well at 37°C for 2 h. The absorbance was measured at a wavelength of 450 nm (Thermo Fisher Scientific, Inc.), and then, the data were recorded and analyzed. The results are presented as the mean ± SD. The treatment groups were compared by ANOVA using GraphPad Prism software (version 9.0).

### 2.4 Cell apoptosis assay

Apoptosis was evaluated according to a previously described protocol (23). An annexin V-FITC/propidium iodide (PI) apoptosis detection kit was purchased from BIO-BOX Biotech (Nanjing, China, BA11100). The cells were treated with various concentrations of xanthatin for 24 h, and then, 1 × 10^6^ cells were collected, washed with prechilled PBS, and resuspended in 500 μL of binding buffer. Then, each sample was incubated with 5 μL of annexin V-FITC and 5 μL of PI for 15 min in the dark at room temperature. Then, the cells were analyzed in a FAC Scan flow cytometer (Becton–Dickinson, San Jose, CA, United States). Data analysis was performed using Flow Jo software (version 7.2.2; TreeStar, Inc. San Carlos, CA, United States).

### 2.5 Western blot analysis

Whole-cell protein lysates were prepared and analyzed by western blotting according to a previously described protocol (23). After being harvested and rinsed with prechilled PBS, the cells were lysed, and the extract was centrifuged at 13,200 × g at 4°C for 15 min. Whole–cell protein lysates (40 μg) were electrophoresed on 12% denaturing polyacrylamide slab gels and transferred to PVDF membranes (BioRad, 1620177) through electroblotting. The membranes were blocked with 5% skim milk for 1 h at room temperature and then probed with specific primary antibodies and subsequently with secondary antibodies. Antibody binding was detected using an ECL system (EMD Millipore, Billerica, MA, United States) according to the manufacturer’s protocol.

### 2.6 Plasmid transfection

The pENTER-c-FLIP plasmid and the control pENTER plasmid were obtained from Biosune Biotechnology Co., Ltd. (Shanghai, China). Y79 cells and WERI-RB-1 cells were seeded in 6-well plates and transfected with the pENTER or pENTER-c-FLIP plasmids, respectively, using X-treme GENE HP DNA Transfection Reagent (Roche Molecular Biochemicals, Mannheim, Germany, 30767600) according to the manufacturer’s protocol. Then, the cells were treated with xanthatin at the indicated concentration for 24 h and subjected to further analysis.

### 2.7 ROS measurement

A Reactive Oxygen Species Assay Kit (Applygen, Beijing, China, C1300-1) with 2′,7′-dichlorodihydrofluorescein diacetate (DCFH-DA) was used to evaluate intracellular ROS based on a method published previously (24). Briefly, after the indicated treatment, the cells were harvested, washed with PBS twice, and incubated with DCFH-DA (0.5 μM) in the dark for 30 min at 37°C. After washing with PBS, the samples were analyzed for DCF fluorescence by BD FACS Verse (BD Biosciences, San Jose, CA, United States).

### 2.8 *In vivo* tumorigenesis analysis

The experiments were performed according to the Regulations for the Administration of Affairs Concerning Experimental Animals and were approved by the Experimental Animal Ethics Committee of the Second Hospital of Shandong University. Nude mice were purchased from Jinan Pengyue Experimental Animal Company (License Number SCXK 2022 0006). They were kept in the animal facility of Shandong University Second Hospital, where bedding was changed every other day, and food and water were provided daily. Four nude mice were housed per cage to ensure sufficient social interaction. At the end of the study, the mice were euthanized by cervical dislocation. Ten five-week-old male athymic nude mice were subcutaneously injected in the left flank region with 200 μL of 3 × 10^7^ Y79 cells mixed with Matrigel (vol/vol, 1:1). After 1 week, successfully transplanted mice were randomly divided into a normal saline group and a xanthatin group. The xanthatin group was treated with xanthatin (0.2 mg/kg; i.p. daily), and the control group was treated with normal saline for 15 days. Tumor sizes and weights were measured every 3 days. Tumor volumes were calculated with the following formula: length × width^2^/2. The tumors were harvested 15 days after injection and stored at −80°C.

### 2.9 Immunohistochemical analysis

Immunohistochemical (IHC) analysis was performed according to a previously described protocol (25). Tumor tissues were fixed in 10% neutral formaldehyde (Baibo Biosciences Co., Ltd., Jinan, China). Following proper dehydration, the tissue samples were embedded in paraffin and then cut into 4-μm-thick sections. After deparaffinization, rehydration, antigen retrieval, and endogenous peroxidase deactivation, the slides were blocked with 10% goat serum and incubated with the corresponding primary antibodies overnight at 4°C. After being washed, the sections were incubated with HRP-conjugated secondary antibody for 45 min at room temperature, and then, the tissues were incubated with 3,3′-diaminobenzidine (DAB) solution and counterstained with hematoxylin. An anti-Ki67 rabbit mAb (Cat No. GB 13030-2) was purchased from Wuhan Service Bio-Technology Co., Ltd. (China).

### 2.10 TUNEL assay

Tissue apoptosis levels were evaluated by a One Step TUNEL apoptosis detection kit (Beyotime, Shanghai, China) according to the manufacturer’s instructions. Tissue samples were sequentially deparaffinized, rehydrated, and permeabilized with 20 μg/mL proteinase K solution for 20 min at 37°C. Then the slices are incubated in a 37°C incubator with Streptavidin-HRP and TBST for 30 min to block endogenous peroxidase. Visualization was performed using a freshly prepared DAB developer. The tissues were photographed under a microscope (Olympus, Tokyo, Japan) after dehydrated and dried after double staining with hematoxylin.

### 2.11 Tissue samples from the GEO database and bioinformatics analysis

The RNA expression data of tissue samples were obtained from the GEO database. The dataset (GSE208143) contained 27 specimens from patients with retinoblastoma and six normal control retina specimens. All the samples were analyzed using Affymetrix Human Genome U133 Plus 2.0 Array. We used the “limma” package in R software to identify the differentially expressed genes (lncRNAs and mRNAs) between retinoblastoma and the control group, with |log2FoldChange (FC)| >1.5 and adjusted *p*-value <0.05. In addition, “dplyr” and “ggplot2” packages were used for data analysis and plot making. “msigdbr,” “ReactomePA,” “tidyverse,” “biomaRt,” “enrich plot,” “clusterProfiler” packages were used for GSEA analysis.

### 2.12 Statistical analysis

All experiments were repeated at least three times under the same controlled conditions. SPSS 20.0 statistical software was used for statistical analysis (IBM Corporation, Armonk, NY, United States). The results are expressed as the mean ± standard deviation of at least three independent assays performed in duplicate or in triplicate. The unpaired *t*-test was used to compare two groups, and one-way ANOVA was used to compare more than two groups. The data were considered significantly different at a *p*-value <0.05. The labels of *, **, ***, and **** respectively indicate the p value < 0.05, *p* value < 0.01, *p* value <0.001, and *p* value <0.0001.

## 3 Results

### 3.1 Xanthatin inhibits the survival of human retinoblastoma cells

**Figure 1:**
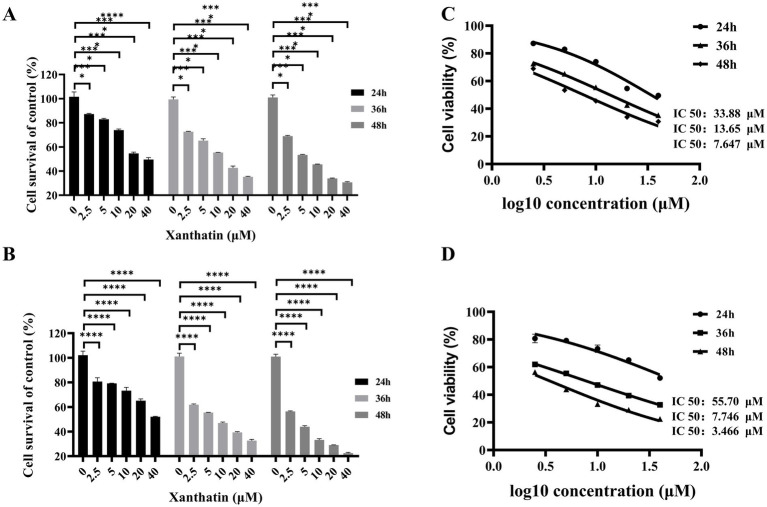
Effects of xanthatin on cell survival of retinoblastoma cell lines Y79 and WERI-RB-1. **(A,B)** Histograms showing cell survival (% of control) of Y79 **(A)** and WERI-RB-1 **(B)** cells treated with varying concentrations of xanthatin (0, 2.5, 5, 10, 20, 40 μM) for 24, 36, and 48 h. Cell survival decreases with increasing xanthatin concentration and treatment duration, with a more pronounced reduction at higher concentrations (20 and 40 μM) and longer time points (48 h). **(C,D)** Dose-response curves illustrating the cell survival (% of control) of Y79 **(C)** and WERI-RB-1 **(D)** cells treated with xanthatin at different concentrations (0 to 2.0 μM) over 24, 36, and 48 h. Cell survival decreases progressively with increasing xanthatin concentration, with a steeper decline at 48 h compared to 24 h and 36 h.

To investigate the cytotoxicity of xanthatin in retinoblastoma cells, Y79 and WERI-RB-1 cells were treated with various concentrations of xanthatin at different times. The effect of xanthatin on cell viability was analyzed by the CCK8 assay. As shown in Figures 1A,B, xanthatin inhibited the survival of retinoblastoma cells in a dose-and time-dependent manner.

The IC50 values were calculated using GraphPad 9.0 software. For Y79 cells, the IC50 values of xanthatin at 24 h, 36 h, and 48 h were 55.7 μM, 7.746 μM, and 3.466 μM, respectively, and for WERI-RB-1 cells, the values were 33.88 μM, 13.65 μM, and 7.647 μM, respectively (Figures 1C,D). These findings suggest that xanthatin effectively suppressed the survival of human retinoblastoma cells.

### 3.2 Xanthatin induces apoptosis in human retinoblastoma cells

**Figure 2:**
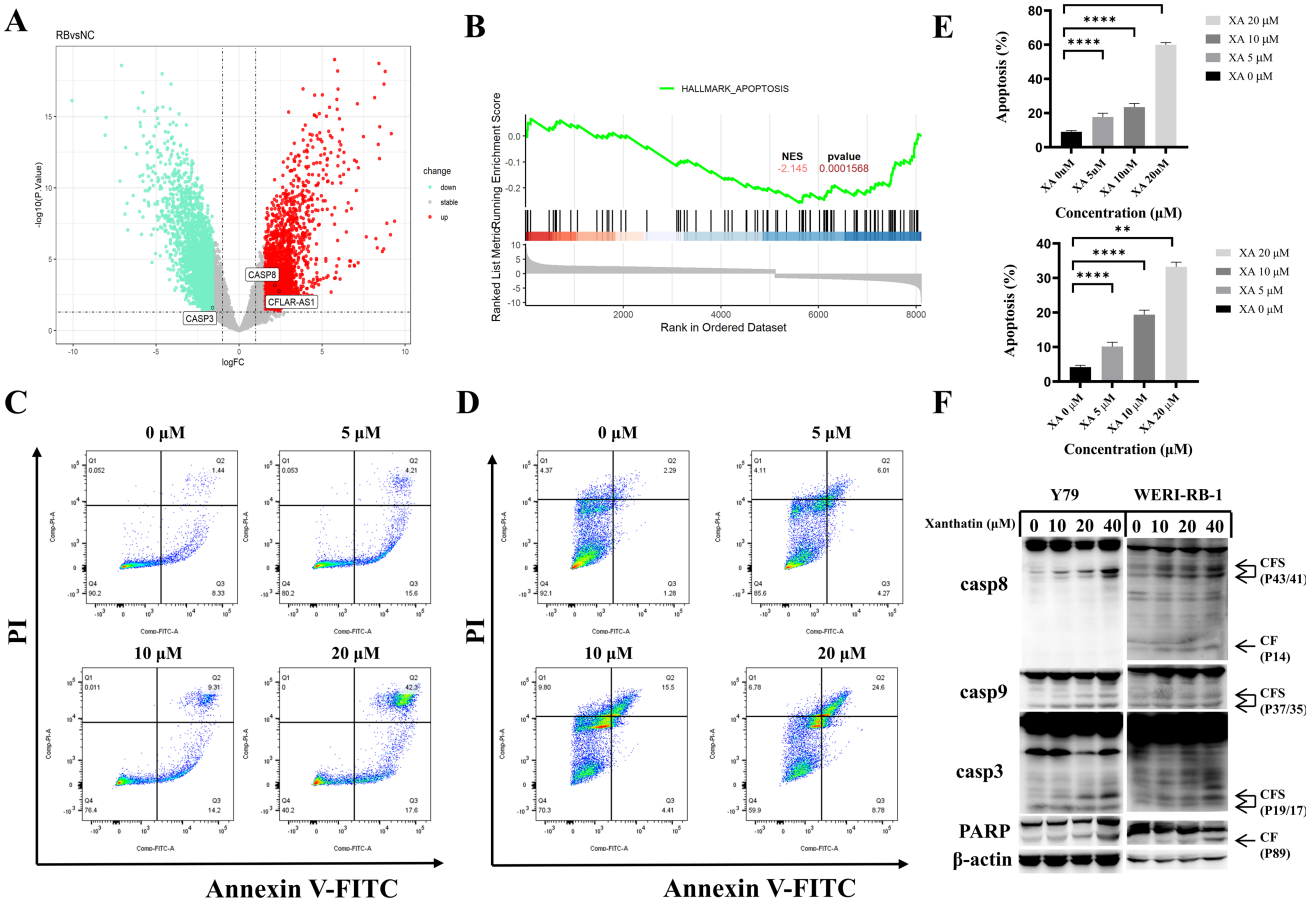
Xanthatin induces caspase-dependent apoptosis in a concentration-dependent manner in retinoblastoma cells. **(A)** Gene expression analysis from the GEO dataset (GSE208143) comparing the retinoblastoma (RB) group to the normal control (NC) group, showed a decrease in caspase-3 expression and an increase in caspase-8 and c-FLIP expression in the RB group. **(B)** Gene Set Enrichment Analysis (GSEA) from GSE208143, highlighting enrichment of the apoptosis gene set in the RB group, with a normalized enrichment score (NES) of 2.145 and a *p*-value <0.01. **(C,D)** Flow cytometry analysis of apoptosis in Y79 **(C)** and WERI-RB-1 **(D)** cell lines treated with xanthatin at concentrations of 0, 5, 10, and 20 μM for 48 h. **(E)** Quantification of apoptosis rates in Y79 and WERI-RB-1 cells treated with xanthatin (0, 5, 10, 20 μM) for 48 h, corresponding to the flow cytometry results in **C,D**. **(F)** Western blot analysis showing the expression levels of caspase-8, caspase-9, caspase-3, cleaved caspase-3, and cleaved PARP in Y79 and WERI-RB-1 cells treated with xanthatin (0, 5, 10, 20 μM).

Based on data from the GEO database and GSEA analysis (Figures 2A,B), we found that in retinoblastoma, caspase-3 is downregulated, while caspase-8 and c-FLIP (cFLAR) are upregulated. The upregulated caspase-8, however, is inhibited by c-FLIP, preventing its binding and activation of caspase-3. This blockage of the apoptotic pathway allows cells to escape apoptosis and proliferate uncontrollably. To determine whether apoptosis was involved in the xanthatin-induced inhibition of cell survival, Y79 cells and WERI-RB-1 cells were treated with xanthatin for 24 h, and cell apoptosis was assessed by PI/FITC-annexin V double staining. Our data revealed that xanthatin had a significant dose-dependent effect on the induction of apoptosis in retinoblastoma cells (Figures 2C–E). When treated with xanthatin (0–20 μM), the frequency of apoptosis increased from 9.77 to 59.9% in Y79 cells and from 3.57 to 33.38% in WERI-1 cells. Furthermore, the expression of apoptotic cleavage proteins was analyzed. Western blot results showed that the expression levels of caspase-8, caspase-9, caspase-3, and cleaved PARP increased in a concentration-dependent manner after the treatment of retinoblastoma cells with xanthatin (Figure 2F). These findings indicate that treatment with xanthatin triggers apoptosis in human retinoblastoma cells.

### 3.3 c-FLIP acts as an anticancer regulator in xanthatin-induced apoptosis

**Figure 3:**
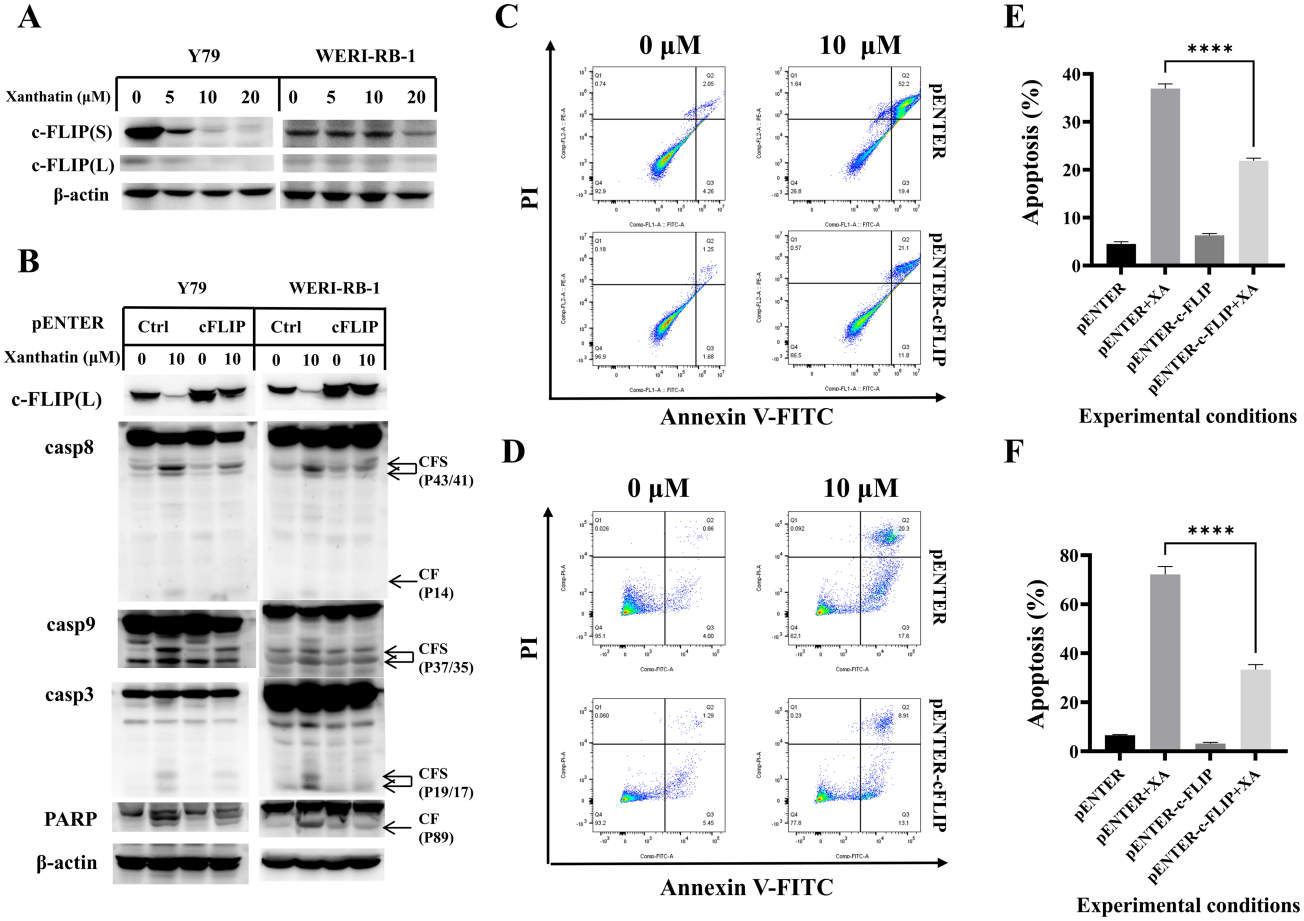
c-FLIP contributes to xanthatin-induced apoptosis. **(A)** Y79 and WERI-RB-1 cells were treated with 0, 5, 10, or 20 μM xanthatin for 24 h and then harvested to test the level of c-FLIP. **(B)** Y79 and WERI-RB-1 cells were transfected with pENTER or pENTER-cFLIP-FALG plasmids. After 48 h of transfection, the cells were exposed to 10 μM xanthatin for 24 h and then harvested to test the expression of cFLIP and apoptosis-related markers (caspase-8 caspase-9, caspase-3, and PARP). **(C)** Flow cytometry of Y79 cells treated with 10 μM xanthatin for 24 h, corresponds to **B**. **(D)** Flow cytometry of WERI-RB-1 cells treated with 10 μM xanthatin for 24 h, corresponds to **B**. **(E,F)** Data analysis was performed using FlowJo software and SPSS software. All data are presented as the mean ± SD.

c-FLIP has been reported to contribute to chemotherapy-induced apoptosis in many types of tumor cells. We found that c-FLIP expression was downregulated in a dose-dependent manner in human retinoblastoma cells following treatment with xanthatin (Figure 3A). In addition, the pENTER plasmid or pENTER-c-FLIP plasmid was transfected into Y79 and WERI-RB-1 cells to confirm whether c-FLIP downregulation played a role in xanthatin-induced apoptosis. As shown in Figure 3B c-FLIP (L) overexpression inhibited the xanthatin-induced cleavage of caspase-8, caspase-9, caspase-3, and PARP. Flow cytometry analysis showed that c-FLIP overexpression reduced xanthatin-induced human retinoblastoma cell apoptosis (Figures 3C,D). In summary, the data demonstrated that c-FLIP has an anticancer effect in xanthatin-induced retinoblastoma cell apoptosis.

### 3.4 Intracellular ROS accumulation is pivotal in xanthatin-induced apoptosis

**Figure 4:**
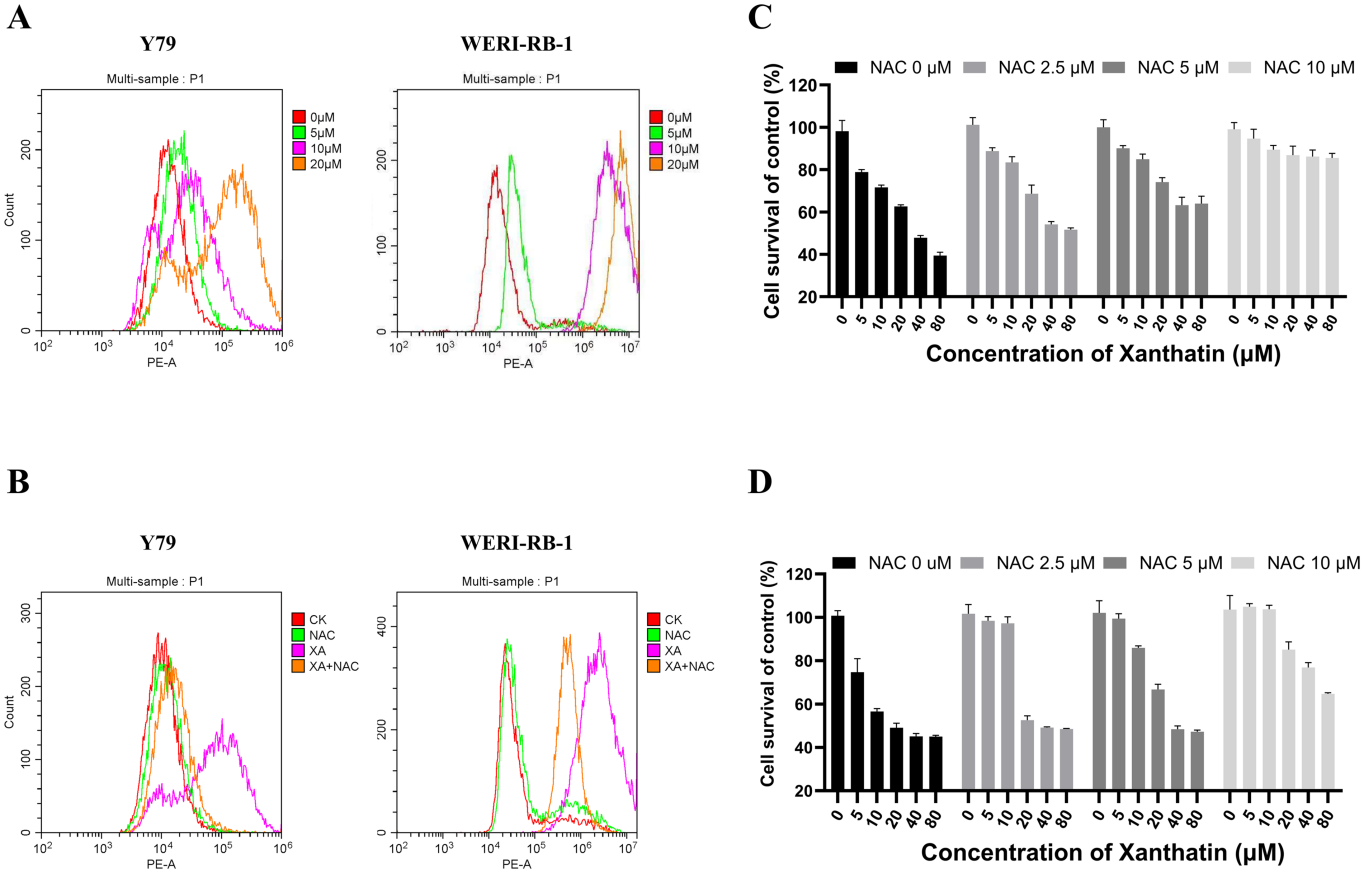
Xanthatin increases ROS production in retinoblastoma cells in a dose-dependent manner, and NAC weakens ROS production and survival inhibition induced by xanthatin. Xanthatin can increase ROS production in RB cells in a dose-dependent manner, and NAC can protect RB cells by reducing ROS production through antioxidation. **(A)** Y79 and WERI-RB-1 cells were treated with 0, 5, 10, or 20 μM xanthatin for 8 h and then harvested for flow cytometry to test the level of ROS. **(B)** Y79 and WERI-RB-1 cells were pretreated with NAC (5 mM) for 2 h before exposure to xanthatin for 8 h. Then, the intracellular ROS levels were measured by flow cytometry. **(C,D)** Flow cytometry of ROS levels in Y79 and WERI-RB1 cells pretreated with NAC (0, 2.5, 5, 10 μM) for 2 h, then treated with 10 μM xanthatin for 24 h.

**Figure 5:**
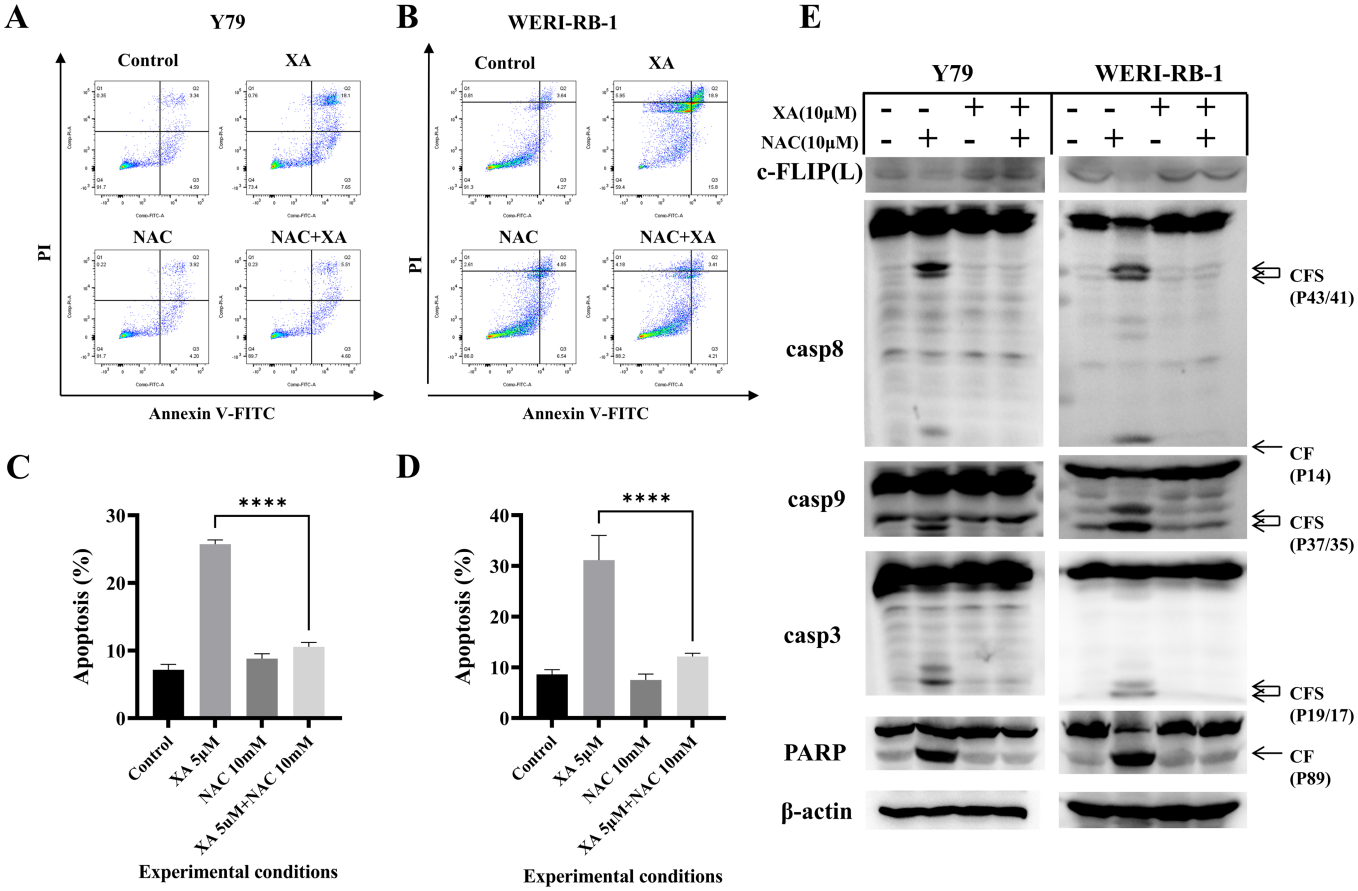
NAC can attenuate xanthatin-induced apoptosis in retinoblastoma cells by reducing ROS production. Y79 and WERI-RB-1 cells were pretreated with NAC (5 mM) for 2 h before exposure to xanthatin for another 24 h. Then, cell apoptosis was measured by flow cytometry **(A,B)**. **(C,D)** Visualized the results of **A,B**. Western blot of apoptosis markers in Y79 and WERI-RB-1 cells pretreated with NAC (0 or 10 μM) for 2 h, then treated with xanthatin (0 or 10 μM) for 24 h. Xanthatin decreased c-FLIP(L) expression and increased cleavage of caspase-8, caspase-9, caspase-3, and PARP, while NAC attenuated these effects **(E)**.

To determine whether ROS were involved in xanthatin-induced retinoblastoma cell apoptosis, we analyzed intracellular ROS generation in retinoblastoma cells after xanthatin treatment. As shown in Figure 4, xanthatin elevated intracellular ROS levels in Y79 and WERI-RB-1 cells in a dose-dependent manner. Then, retinoblastoma cells were pretreated with NAC, an antioxidant. We found that NAC combined with xanthatin, compared to xanthatin treatment alone, decreased the levels of ROS generation in Y79 and WERI-RB-1 cells (Figure 4B). Afterwards, CCK8 was added to the cells to test cell viability (Figures 4C,D) and shown that NAC markedly attenuated xanthatin-induced survival inhibition, suggesting that ROS were involved in the antitumor effect of xanthatin. Furthermore, we tested whether the xanthatin-induced apoptosis of retinoblastoma cells is affected by NAC. Y79 cells and WERI-RB-1 cells were treated with different concentrations of NAC for 2 h and then treated with different concentrations of xanthatin for another 24 h. Apoptosis was measured by flow cytometry (Figures 5A–D). NAC markedly attenuated xanthatin-induced apoptosis. Moreover, compared to those with xanthatin treatment alone, NAC combined with xanthatin decreased the expression levels of c-FLIP (L) and cleaved caspase-8, caspase-9, caspase-3, and PARP (Figure 5E). Therefore, we concluded that xanthatin triggers ROS-mediated c-FLIP downregulation, which leads to cell apoptosis.

### 3.5 Xanthatin triggers endoplasmic reticulum stress in retinoblastoma cells

**Figure 6:**
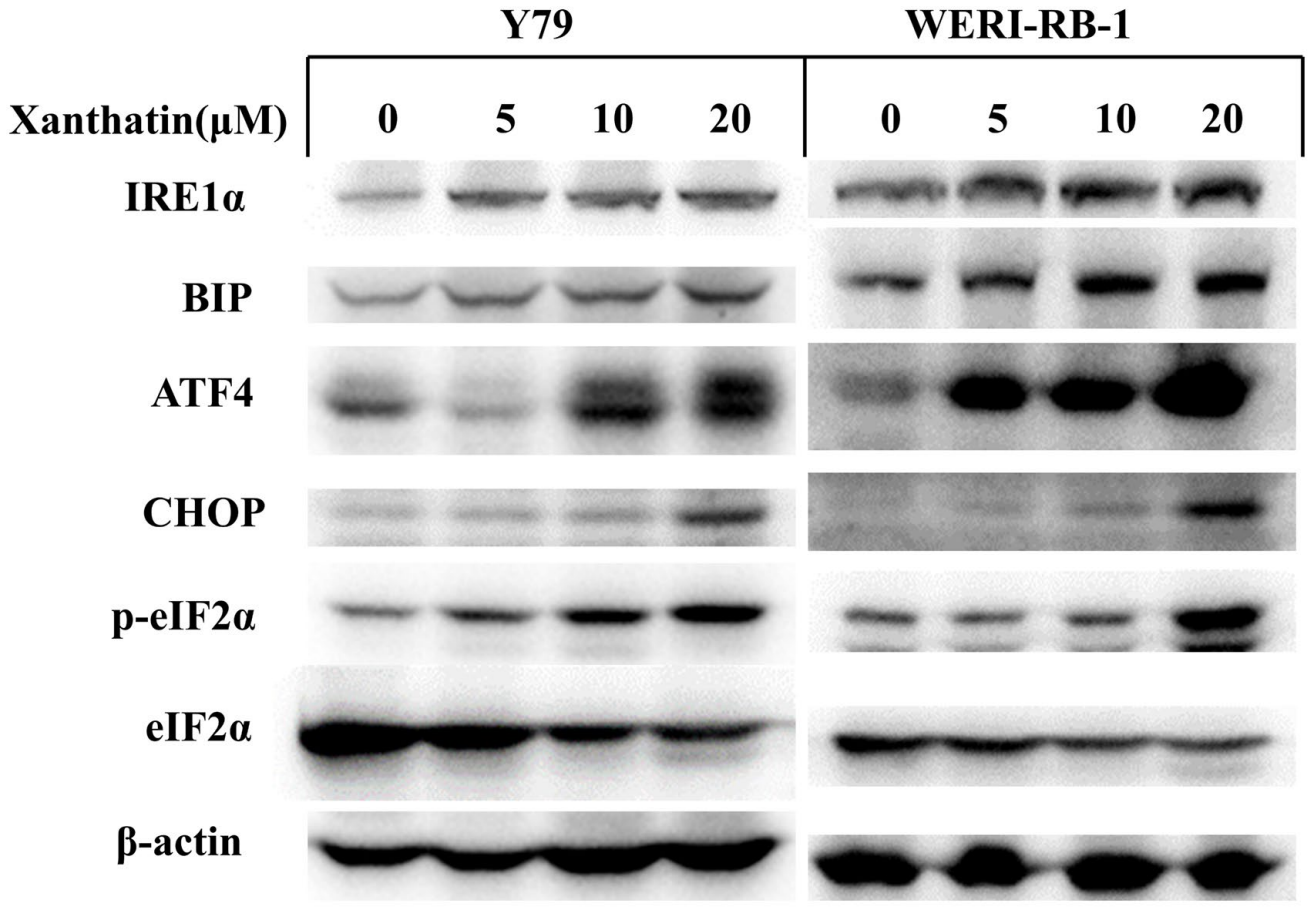
Xanthatin triggers endoplasmic reticulum stress in human retinoblastoma cells. Xanthatin triggers endoplasmic reticulum stress in human retinoblastoma cells. Y79 and WERI-RB-1 cells were treated with 0, 5, 10, and 20 μM xanthatin and incubated for 24 h. Following treatment, endoplasmic reticulum stress-related proteins were quantified by western blotting analysis.

The endoplasmic reticulum (ER) response is activated when cancer cells are treated with chemotherapeutics. We examined relevant proteins in the ER stress pathway to confirm whether xanthatin triggers ER stress in retinoblastoma cells. The western blot results demonstrated that the expression of the marker proteins IRE1α, BiP, p-eIF2α, ATF4, and CHOP was upregulated in a dose-dependent manner in retinoblastoma cells after xanthatin treatment (Figure 6). These data indicate that xanthatin triggers ER stress in retinoblastoma cells.

### 3.6 Xanthatin exerted antitumor effects in Y79 xenograft models

**Figure 7:**
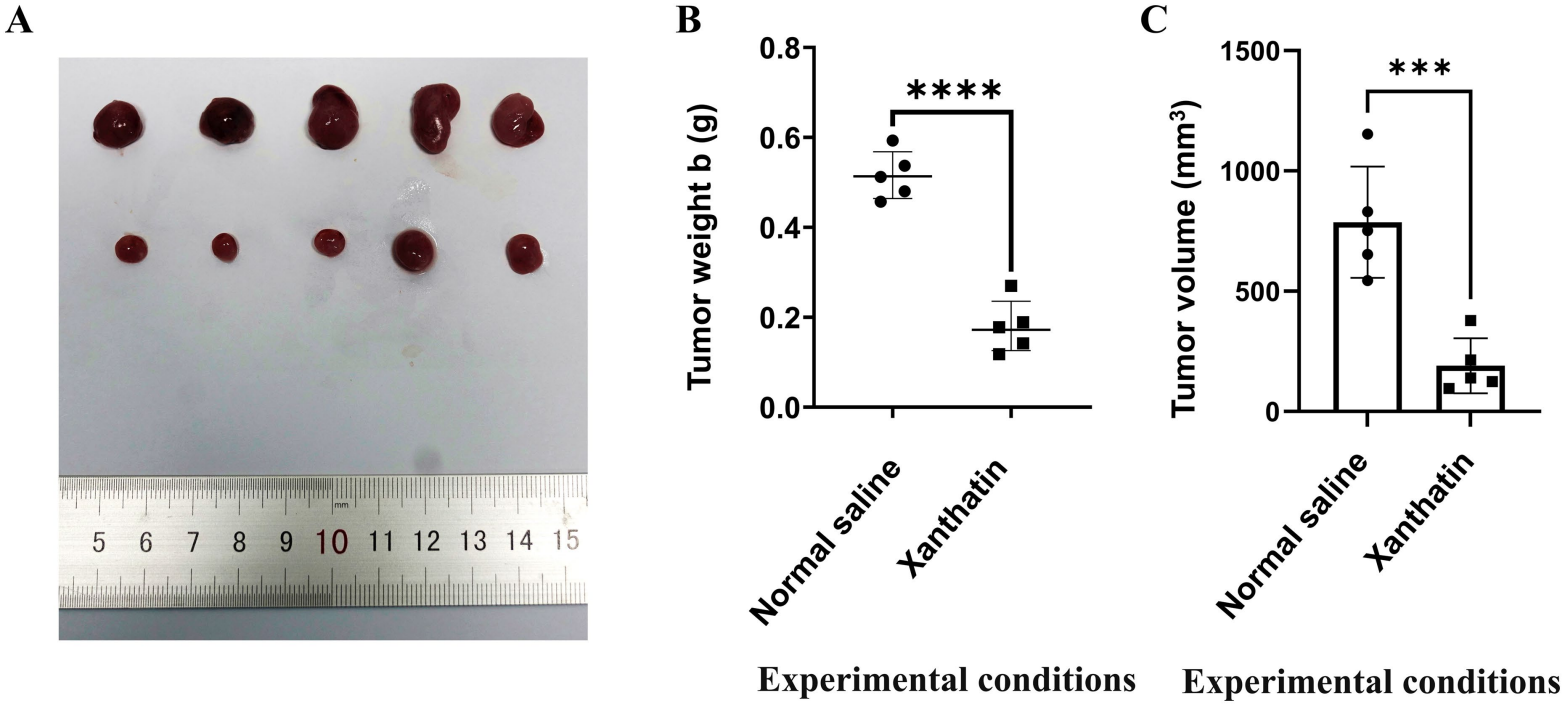
Xanthatin inhibits human retinoblastoma cell tumorigenesis *in vivo*. **(A)** The xenograft tumors collected from the BALB/c nude male mice were randomly divided into a xanthatin-treaded group and a normal saline group after subcutaneously injected with Y79 cells for 15 days. **(B)** Tumor weight of mice treated with normal saline or xanthatin. Mean ± SD (*n* = 5). ^***^p < 0.001 vs. normal saline. **(C)** Tumor volume of mice treated with normal saline or xanthatin. Mean ± SD (*n* = 5). ^***^p < 0.001 vs. normal saline.

**Figure 8:**
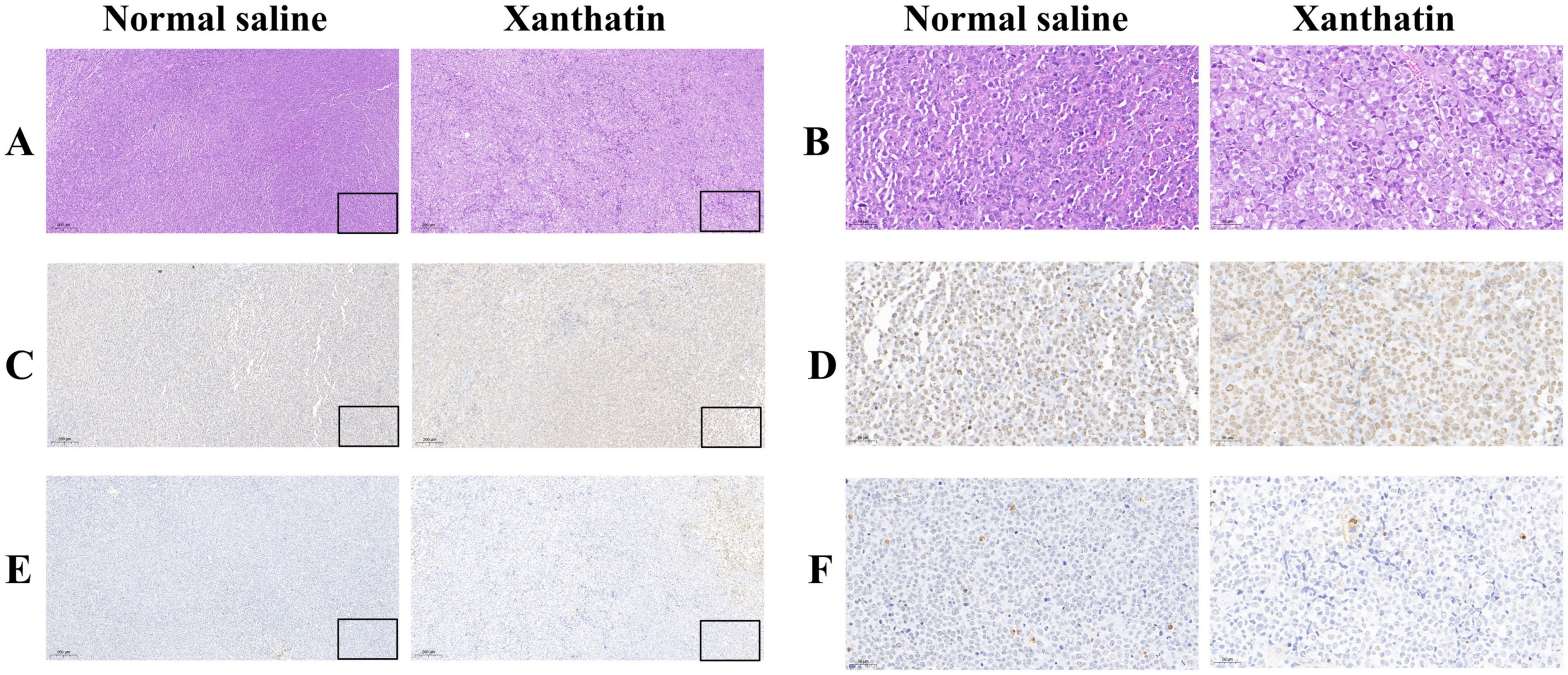
Xanthatin-induced retinoblastoma cell apoptosis *in vivo*. Scale bars = 100 μm. **(A,B)** H&E staining of tumor sections from mice treated with normal saline or xanthatin. Xanthatin reduced tumor cell density. **(C,D)** IHC staining of tumor sections from mice treated with normal saline or xanthatin, showing decreased expression of a proliferation marker in the xanthatin group. **(E,F)** IHC staining of tumor sections from mice treated with normal saline or xanthatin, showing increased expression of an apoptosis marker in the xanthatin group. Magnification: ×40. Corresponding scale bars are depicted in the lower right corner of each image. Scale bars = 100 μm.

We constructed Y79 xenograft models to confirm the antitumor effect of xanthatin *in vivo*. As shown in Figure 7, there was a significant reduction in tumor volume and weight in xanthatin-treated mice compared to control mice on the 15th day. The results of HE analysis showed that there were more necrotic cells in the xanthatin-treated group than in the saline-treated group (Figures 8A,B). Furthermore, the results of proliferation marker (Ki67) analysis showed an obvious decrease in Ki67 expression in the tumors of mice treated with xanthatin compared with the tumors of mice treated with normal saline (Figures 8C,D). TUNEL staining showed that the number of apoptotic (TUNEL positive) cells were significantly higher in the tumors of xanthatin-treated mice than in the tumors of control mice (Figures 8E,F). These results were consistent with the *in vitro* results and indicated that xanthatin was an efficacious antitumor drug in the xenograft model.

## 4 Discussion

Retinoblastoma, derived from the developing retina, accounts for 3% of all childhood cancers (26). Therefore, the identification of novel adjuvant treatments for retinoblastoma is still needed.

Traditional Chinese medicine (TCM) has been used for thousands of years in China and East Asia (27). Xanthatin is a well-known traditional herbal medicine in China and Vietnam (4). Recent studies have shown the powerful antitumor effect of xanthatin in different cancers (7–11). A previous study found that xanthatin exerted its pharmacological effects by inhibiting the PLK1-mediated G2/M pathway and inducing retinoblastoma cell apoptosis (12). However, the underlying mechanism and the antitumor effect *in vivo* require further research. In the present study, we investigated whether xanthatin exhibited antitumor effects in retinoblastoma cells and explored the underlying molecular mechanism.

Most anticancer therapies trigger apoptosis and related cell death networks to eliminate malignant cells (28). Similar to previous studies, we found that xanthatin induced cell apoptosis, including both early apoptosis and late apoptosis, in retinoblastoma cells, with significant upregulation of the cleavage of caspase-8, -9, -3, and PARP. In conclusion, xanthatin exerts anticancer effects by decreasing cell viability and inducing caspase-dependent apoptosis in retinoblastoma cells.

c-FLIP is a major antiapoptotic regulator that is recruited to the DISC and regulates the activation of caspases-8 and -10 in the death receptor signaling pathways (29). However, whether xanthatin inhibits tumor cells by downregulating the expression of c-FLIP has not been reported. We found that the protein levels of both c-FLIP(L) and c-FLIP(S) were reduced, in a concentration-dependent pattern, in retinoblastoma cells after xanthatin. Enhancing c-FLIP (L) expression decreases xanthatin-induced apoptosis in Y79 and WERI-RB-1 cells. These data suggest that decreased c-FLIP expression plays a pivotal role in the induction of apoptosis by xanthatin.

Importantly, our study further indicated that xanthatin-induced cell apoptosis was related to ROS. It has been reported that the regulation of the intracellular redox status by ROS is related to apoptosis by influencing intracellular signaling pathways (22, 30). In the present study, we noted that in retinoblastoma cells, the accumulation of intracellular ROS was triggered by xanthatin. In addition, the ROS scavenger NAC exerted strong effects not only by decreasing xanthatin-induced intracellular ROS and apoptosis but also by attenuating the xanthatin-induced decrease in c-FLIP. Hence, xanthatin induces apoptosis in retinoblastoma cells by upregulating intracellular ROS levels and downregulating c-FLIP expression.

Xanthatin has been reported to induce glioma cell apoptosis and inhibit tumor growth by activating the ER stress-dependent CHOP pathway (31). We examined relevant proteins in the ER stress pathway to confirm whether xanthatin induces ER stress in retinoblastoma cells. The results demonstrated that xanthatin upregulated the expression of the marker proteins IRE1α, BiP, p-eIF2α, ATF4, and CHOP in a concentration-dependent manner. These data indicate that xanthatin triggers ER stress in retinoblastoma cells. However, whether ER stress is involved in xanthatin-induced ROS and apoptosis requires further research.

To further investigate whether xanthatin could affect tumorigenesis *in vivo*, a Y79 xenograft model was established, and the results verified the pro-apoptotic effect of xanthatin *in vivo* in mice. The tumor volume and tumor weight of mice treated with xanthatin were lower than those of control mice. Furthermore, H&E staining and IHC analysis showed that there were many necrotic cells in the xanthatin treatment group, with no obvious necrosis in the control group. Moreover, compared to the xanthatin-treated group, in the normal saline-treated group, there were numerous Ki67-stained cells, and TUNEL staining showed more apoptotic tissue in the xanthatin treatment group. In summary, our results indicated that xanthatin could prevent xenograft tumor growth *in vivo*.

In conclusion, our findings demonstrate that xanthatin exerts pro-apoptotic effects on retinoblastoma cells. An increase in ROS and the downregulation of c-FLIP contribute to the antitumor effect of xanthatin in retinoblastoma cells. Moreover, xanthatin inhibits retinoblastoma cell tumorigenesis *in vivo*. The results of this study provide an important theoretical basis for promoting xanthatin as a potential clinical therapeutic strategy for the treatment of human retinoblastoma.

## Data Availability

Publicly available datasets were analyzed in this study. This data can be found at: https://www.ncbi.nlm.nih.gov/geo/query/acc.cgi?acc=GSE208143.
